# Activation of CREBZF Increases Cell Apoptosis in Mouse Ovarian Granulosa Cells by Regulating the ERK1/2 and mTOR Signaling Pathways

**DOI:** 10.3390/ijms19113517

**Published:** 2018-11-08

**Authors:** Fenglei Chen, Xin Wen, Pengfei Lin, Huatao Chen, Aihua Wang, Yaping Jin

**Affiliations:** 1 Department of Clinical Veterinary Medicine, College of Veterinary Medicine, Northwest A&F University, Yangling 712100, Shaanxi, China; flchen@yzu.edu.cn (F.C.); wenx199288@163.com (X.W.); linpengfei@nwsuaf.edu.cn (P.L.); htchen@nwsuaf.edu.cn (H.C.); aihuawang1966@163.com (A.W.); 2 Key Laboratory of Animal Biotechnology of the Ministry of Agriculture, Northwest A&F University, Yangling 712100, Shaanxi, China; 3 Department of Basic Veterinary Medicine, College of Veterinary Medicine, Yangzhou University, Yangzhou 225009, Jiangsu, China; 4 Jiangsu Co-Innovation Center for Prevention and Control of Important Animal Infectious Diseases and Zoonoses, Yangzhou 225009, Jiangsu, China

**Keywords:** apoptosis, granulosa cells, mTOR, ERK1/2

## Abstract

CREBZF, a multifunction transcriptional regulator, participates in the regulation of numerous cellular functions. The aims of the present study were to detect the localization of CREBZF expression in the ovary and explore the role of CREBZF and related mechanisms in the apoptosis of ovarian granulosa cells. We found by immunohistochemistry that CREBZF was mainly located in granulosa cells and oocytes during the estrous cycle. Western blot analysis showed that SMILE was the main isoform of CREBZF in the ovary. The relationship between apoptosis and CREBZF was assessed via CREBZF overexpression and knockdown. Flow cytometry analysis showed that CREBZF induced cell apoptosis in granulosa cells. Western bolt analysis showed that overexpression of CREBZF upregulated BAX and cleaved Caspase-3, while it downregulated BCL-2. Furthermore, overexpression of CREBZF inhibited the ERK1/2 and mTOR signaling pathways through the phosphorylation of intracellular-regulated kinases 1/2 (ERK1/2) and p70 S6 kinase (S6K1). Moreover, we found that CREBZF also activated autophagy by increasing LC3-II. In summary, these results suggest that CREBZF might play a proapoptotic role in cell apoptosis in granulosa cells, possibly by regulating the ERK1/2 and mTOR signaling pathways.

## 1. Introduction

In mammals, the ovary is an extremely dynamic organ, which consists of ovarian follicles at various stages of development and of corpora lutea. The ovarian follicle develops through the primordial, primary, secondary, and antral stages [1]. However, only very few follicles reach the ovulatory stage and subsequently form the corpus luteum (CL), and most of them undergo atresia [2]. Numerous research studies have identified not only endocrine but also paracrine and autocrine factors, which include gonadal steroids, growth factors, cytokines, and intracellular proteins, that play important roles in ovarian follicular development [3]. For example, an increasing body of evidence indicates that members of the mammalian basic leucine zipper (bZIP) transcription factor superfamily regulate the key genes that are crucial for ovulation [4]. It is well demonstrated that a complex series of cellular and molecular events are initiated during follicular development, atresia, ovulation, and subsequently luteinization of the postovulatory follicle [5,6,7,8]. These processes require the precisely regulated expression of a complex, interacting network of genes, the expression of many of which is initiated by bZIP family members [9,10,11].

CREBZF is a member of the cAMP response element-binding protein (CREB)/activating transcription factor (ATF) family of the bZIP transcription factors, which was identified through its interaction with herpes simplex virus-1 (HSV-1)-related host cell factor 1 (HCF-1) [12,13]. Previous studies have demonstrated that CREBZF has two isoforms derived from a differential usage of the initiation codons, including SMILE (long isoform of CREBZF) and Zhangfei or ZF (short isoform of CREBZF) [13]. To date, it has been reported that CREBZF is a multifunctional transcription factor or regulator. As an inhibitor, CREBZF suppresses the replication of HSV by inhibiting the activation of VP16 [12], regulates endoplasmic reticulum (ER) stress by inhibiting unfolded protein response (UPR)-related bZIP proteins such as Luman, XBP1, and ATF6 [14,15], acts as a corepressor of estrogen receptor and other nuclear receptors (NRs) [13,16,17], inhibits the secretion of insulin, resulting in glucolipotoxicity in INS-1 rat insulinoma cells [18,19], and inhibits SMADs related to the BMP and TGF-β signaling pathways by binding SMAD8 [20]. As an activator, CREBZF binds ATF4 in response to the activation of the mitogen-activated protein kinases (MAPK) signaling pathway by MEK1 [21], participates in tumor suppression by activating p53 [22,23], and induces cellular differentiation and cell apoptosis by activating the TrkA–NGF–MAPK signaling pathway in ONS-76 medulloblastoma cells [24]. In addition to apoptosis induction, CREBZF appears to induce autophagy by upregulating the expression of autophagy response genes (ATG), such as ATG5, ATG6, and ATG7 [24].

Recently, a study reported that CREBZF plays a potential role in mediating uterine receptivity during the implantation and development of preimplantation embryos in mice [25,26]. Moreover, CREBZF is regulated by activating blastocysts and estrogen (E2) in mouse uteri, and SMILE is the main isoform of CREBZF in this process [25]. We speculated that CREBZF may cooperate in the regulation of reproductive functions. A previous study demonstrated that CREBZF, with another transcription factor, leads to differentiation, apoptosis, and autophagy in other cells [24]. All of these processes occur in granulosa cells during follicular development and atresia. Hence, we set out to know whether CREBZF cooperates in these processes and which CREBZF isoform plays the main role. We determined the localization of CREBZF in the mouse ovary in vivo, the role of CREBZF in ovarian granulosa cells apoptosis in vitro, and its mechanism.

## 2. Results

### 2.1. Expression of CREBZF in the Ovaries throughout the Estrous Cycle

**Figure 1:**
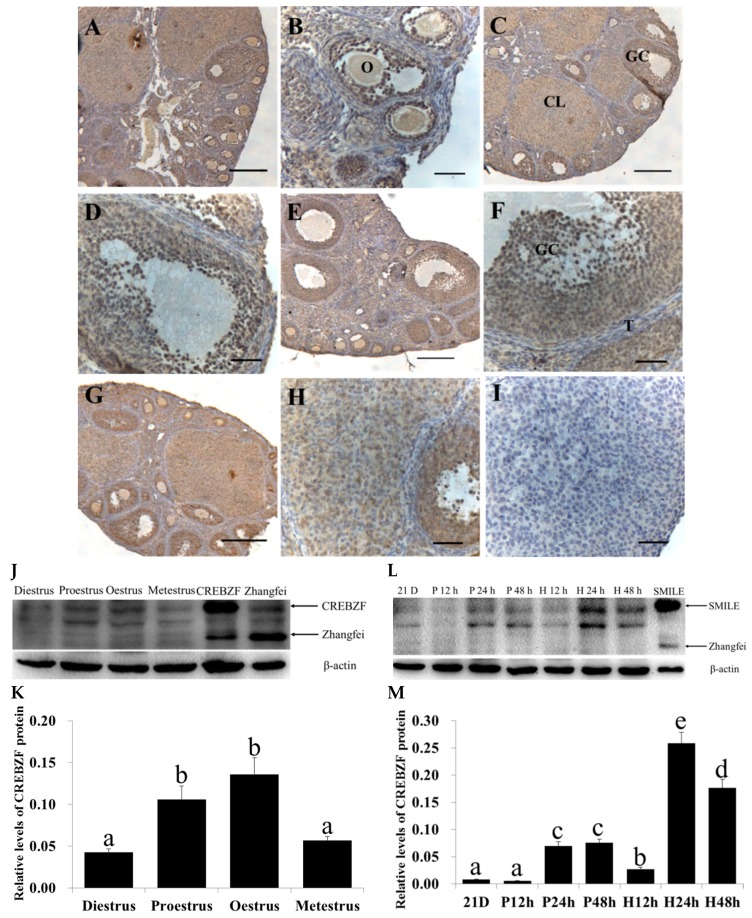
Localization and expression of CREBZF protein in the mouse ovaries during the estrous cycle. (**A**,**B**) Diestrus; (**C**,**D**) proestrus; (**E**,**F**) oestrus; (**G**,**H**) metestrus; (**I**) Negative control. Positive immunostaining for CREBZF is indicated by a brown reaction product. GC, granulosa cells; O, oocyte; T, theca cells; CL, corpus luteum. Scale bars, 100 μm (**A**,**C**,**E**,**G**) and 50 μm (**B**,**D**,**F**,**H**,**I**). (**J**,**K**) Protein levels of SMILE and Zhangfei in the ovary during the estrous cycle; (**L**,**M**) protein levels of SMILE and Zhangfei in the ovary after treatment with pregnant mare serum gonadotropin (PMSG) or human chorionic gonadotropin (hCG); 21 D, 21-day-old; P12h, P24h, and P48h, treated with PMSG for 12, 24 and 48 h, respectively; H12h, H24h, and H48h, after treatment with PMSG for 48 h and hCG for 12, 24 and 48 h, respectively. Analyses of the band intensity on films are presented as the relative ratio of SMILE or Zhangfei to β-actin. Statistical analysis is shown in the bar graphs. Data are presented as the mean ± SEM. The bars with different letters indicate significant differences (*p* < 0.05), while the bars with the same letter indicate no difference between their respective values.

To elucidate whether CREBZF was expressed in the ovaries and which isoform was primarily expressed, we collected the ovaries during four phases of the estrous cycle. Immunohistochemical staining analysis showed that the expression of CREBZF was ubiquitous throughout the ovary during the estrous cycle. In the different follicular development stages, the expression of CREBZF was stronger in the granulosa cells and oocytes at the various stages of the estrous cycle than that in the theca cells and corpus luteum (Figure 1A–I). In addition, positive staining for CREBZF was present in the vestigial granulosa cell layers of atretic follicles (Figure S1). Apoptosis granulosa cells started appearing in early atretic follicles. Furthermore, an increasing number of granulosa cells underwent apoptosis in progressed and late atretic follicles, with a severely distrusted granulosa layer (Figure S1C–E). Morphologically, the apoptotic granulosa cells shrank and reduced their size; especially, their nuclei became condensed and were stained deep blue by hematoxylin. However, the granulosa layer was aligned along the follicular basal lamina, and deep blue-stained nuclei of granulosa cells were not observed in growing healthy follicles (Figure S1A,B). Western blot analysis showed that the primary CREBZF isoform was SMILE, identified by comparison with SMILE- and Zhangfei-overexpressing cells, whereas Zhangfei was almost not detected during the estrous cycle. The expression of SMILE was higher at proestrus and estrous during the estrous cycle (*p* < 0.05, Figure 1J,K). After pregnant mare serum gonadotropin (PMSG) and human chorionic gonadotropin (hCG) treatment of immature mice, we also mainly detected SMILE, and the levels of SMILE in the ovary were increased after 24 h and 48 h of PMSG treatment and were the highest after 24 h of hCG treatment (*p* < 0.05, Figure 1L,M).

### 2.2. Effects of Lentivirus-Induced CREBZF Overexpression and Knockdown in Primary Granulosa Cells 

**Figure 2:**
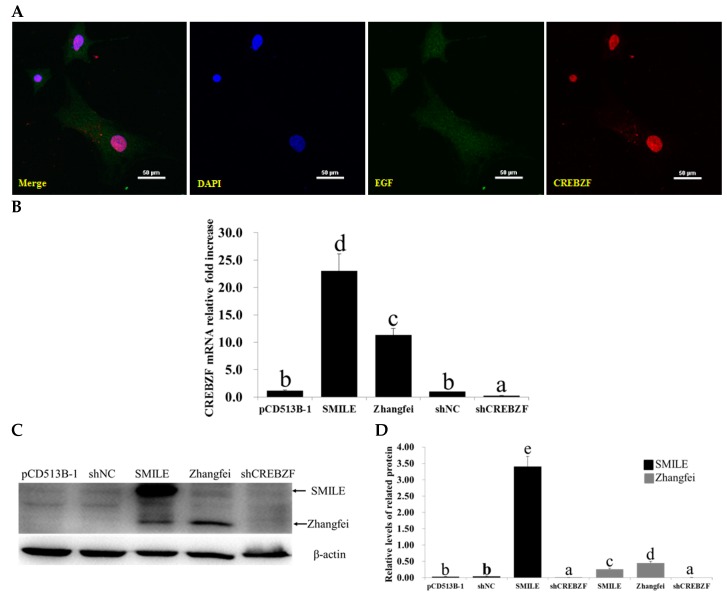
CREBZF expression in primary mouse ovarian granulosa cells after transduction of CREBZF lentiviruses. (**A**) Immunofluorescence analysis of CREBZF expression levels in ovarian granulosa cells transduced with CREBZF lentivirus for 48 h. Scale bars, 50 μm; (**B**) relative mRNA expression of the *CREBZF* gene in ovarian granulosa cells transduced with CREBZF lentivirus for 48 h. The amounts of mRNA were normalized to that of β-actin; (**C**,**D**) western blot analysis of SMILE and Zhangfei expression levels in ovarian granulosa cells transduced with CREBZF lentiviruses for 48 h. The statistical analysis is shown in the bar graphs. Data are presented as the mean ± SEM. The bars with different letters show significant differences (*p* < 0.05), while the bars with the same letter show no difference between their respective values.

To identify the effects of CREBZF overexpression and knockdown, granulosa cells were cultured in vitro. The analysis of the isolated cells showed that almost all adherent cells were specifically stained with anti-aromatase antibody, demonstrating that the primary granulosa cells were pure (Figure S2). The granulosa cells were transduced with CREBZF overexpression and short-hairpin interfering RNAs (shRNAs) lentiviruses (Figure 2A). The transduction efficiency was more than 80% (the results are not shown). Immunofluorescence staining showed that CREBZF was primarily located in the nucleus of the granulosa cells and the CREBZF overexpression lentiviral vector significantly increased the expression of CREBZF. RT-qPCR analysis showed that CREBZF overexpression lentivirus significantly increased SMILE and Zhangfei mRNA levels in granulosa cells (Figure 2B). CREBZF shRNA (shCREBZF) lentivirus markedly decreased the expression of both SMILE and Zhangfei mRNA than that of negative control shRNA (shNC) lentivirus (Figure 2B). Meanwhile, western blot analysis also showed the same results (*p* < 0.05, Figure 2C,D). We found that SMILE overexpression lentivirus significantly increased both SMILE and Zhangfei (Figure 2C).

### 2.3. Effect of CREBZF on Ovarian Granulosa Cells Apoptosis

**Figure 3:**
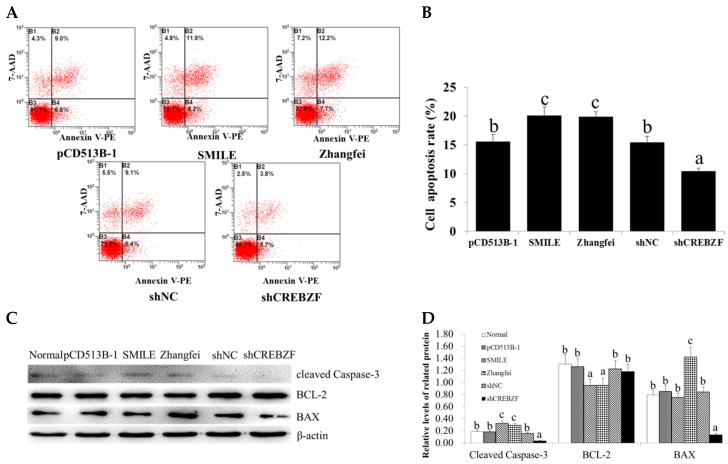
Effects of CREBZF overexpression and knockdown on apoptosis in ovarian granulosa cells. (**A**,**B**) Measurement of cell apoptosis via flow cytometry in granulosa cells transduced with CREBZF lentivirus for 48 h. (**C**,**D**) Expression of cell apoptosis-related genes (BCL-2, BAX, and cleaved Caspase-3) in ovarian granulosa cells transduced with CREBZF lentivirus for 48 h. The statistical analysis is shown in the bar graphs. Data are presented as the mean ± SEM. The bars with different letters indicate significant differences (*p* < 0.05), while the bars with the same letter indicate no difference between their respective values.

To elucidate the roles of CREBZF in the regulation of granulosa cells apoptosis, we determined the apoptotic rate of transduced granulosa cells by Annexin V-PE/7–AAD double staining using flow cytometry. Flow cytometry analysis showed that the overexpression of CREBZF significantly increased cell apoptosis in both the SMILE and the Zhangfei overexpression groups (20.1% and 19.9%, respectively) compared with the control vector group (the pCD513B-1 group, 15.6%) (Figure 3A,B). However, knockdown of CREBZF (10.5%) inhibited granulosa cells apoptosis compared with the shNC group (15.5%) (Figure 3A,B).

To confirm whether CREBZF activated the apoptotic signaling pathway, we detected the expression of apoptosis-related proteins, such as BCL-2, BAX, and cleaved Caspase-3. Western blot analysis showed that the overexpression of CREBZF significantly upregulated BAX and cleaved Caspase-3, while it downregulated BCL-2 in both the SMILE and the Zhangfei overexpression groups (Figure 3C,D). However, knockdown of CREBZF significantly upregulated BCL-2, while it downregulated BAX and cleaved Caspase-3 (Figure 3C,D). Consistent with the western bolt results, the results of qRT-PCR showed that the overexpression of CREBZF increased the expression of BAX and Caspase-3 mRNA, while it decreased BCL-2 mRNA. However, knockdown of CREBZF significantly upregulated BCL-2 mRNA, while it downregulated BAX and Caspase-3 mRNA (Figure S3).

### 2.4. Effect of CREBZF on ERK1/2 and mTOR Signaling Pathways

**Figure 4:**
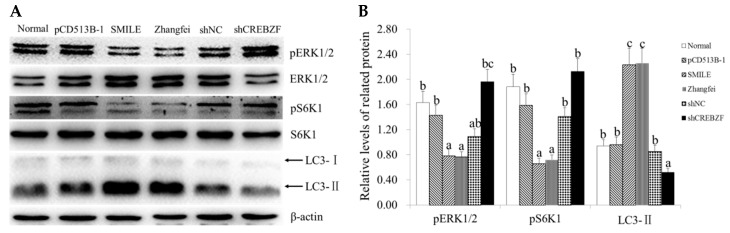
Effects of CREBZF overexpression and knockdown on ERK1/2 and mTOR signaling pathways in ovarian granulosa cells. (**A**,**B**) Expression of ERK1/2, pERK1/2, S6K1, pS6K1, LC3-I. and LC3-II in ovarian granulosa cells transduced with CREBZF lentivirus for 48 h. The statistical analysis is shown in the bar graphs. Data are presented as the mean ± SEM. The bars with different letters indicate significant differences (*p* < 0.05), while the bars with the same letter indicate no difference between their respective values.

To explore the mechanism of CREBZF-induced apoptosis, we detected the expression of ERK1/2 and mTOR signaling pathways-related protein ERK1/2 and S6K1. Western blot analysis showed that the overexpression of CREBZF significantly downregulated the phosphorylation of ERK1/2 and S6K1, while the knockdown of CREBZF significantly upregulated the phosphorylation of ERK1/2 and S6K1 in both the SMILE and the Zhangfei overexpression groups (Figure 4A,B). We also detected the expression of LC3-II, a special marker of autophagy activation. Western blot analysis showed that the overexpression of CREBZF significantly upregulated LC3-II in both the SMILE and the Zhangfei overexpression groups, while the knockdown of CREBZF significantly downregulated LC3-II (Figure 4A,B).

## 3. Discussion

This study is the first to analyze CREBZF expression variations during the estrous cycle. We determined the localization of CREBZF in the ovaries, showing that CREBZF is widespread in the ovarian tissue, especially in granulosa cells and oocytes, and significantly increases during the proestrus and estrous phases. Consistent with a previous study, CREBZF is expressed at high levels in the luminal and glandular epithelium of the uterus at the proestrus and oestrus phases [25,26]. We speculated that CREBZF might participate in follicular development and be regulated by the sex hormones in the reproductive system. CREBZF generates two isoforms, SMILE and Zhangfei, from the alternative usage of initiation codons [13]. However, few studies have demonstrated the different localizations and functions of SMILE and Zhangfei in the ovary. In this study, we demonstrated that SMILE is the main isoform in the ovary. Consistent with our previous study, SMILE is mainly detected in the uterus [25].

In addition, we transduced CREBZF lentiviruses into primary granulosa cells in vitro. We found that activation of CREBZF induced cell apoptosis in ovarian granulosa cells. One of the main mechanisms of apoptosis induction is a mitochondrial signaling cascade, which is mediated by BCL-2 and caspase family members. In this study, we found that activation of CREBZF significantly downregulated BCL-2, while it upregulated BAX and cleaved Caspase-3. Consistent with other studies, we speculated that CREBZF might have a proapoptotic role in ovarian granulosa cells [18,22,24,27,28,29,30]. CREBZF may be a new potential focus of research in the study of the pathophysiological mechanisms of follicular development and atresia.

Furthermore, we detected potential regulatory mechanisms of CREBZF in apoptosis induction in ovarian granulosa cells. ERK1/2 activate cell cycle progression, mediate the mitogenic action of hormones and growth factors, stimulate ovarian cell proliferation, differentiation, and secretion activity [31,32,33,34,35,36,37,38], and suppress cell apoptosis [39,40,41,42]. The ERK1/2 signaling pathway is regulated in the ovary [39]. Agnieszka et al. documented that the ERK1/2 signaling pathway is involved in a follicular antiapoptotic response in pigs [43]. Accordingly, we reasoned that the ERK1/2 signaling pathway might be involved in CREBZF-induced apoptosis in ovarian granulosa cells. In this study, we found that both SMILE and Zhangfei could decrease the phosphorylation of ERK1/2. Bodnarchuk et al. reported that CREBZF activates the expression of Brn3a which, in turn, activates the expression of TrkA. NGF binds TrkA, activating MAPK, JNK signaling, or both, leading to differentiation, apoptosis, and autophagy in ONS-76 cells [24]. Hence, we speculated that CREBZF not only activates but also inhibits MAPK signaling pathways in ovarian granulosa cells.

mTOR is a serine/threonine protein kinase, regulating cell proliferation, tissue growth, and autophagy. mTOR mediates downstream signaling pathways through phosphorylation of S6K1, leading to initiation of protein translation. The mTOR signaling pathway plays a critical role in the regulation of ovarian cell proliferation, apoptosis, secretory activity, and folliculogenesis [44,45,46,47,48]. mTOR inhibition suppresses the proliferation of ovarian granulosa cells [49]. Moreover, mTOR increases in FSH-mediated granulosa cell proliferation via ERK1/2 signaling pathway [49]. In this study, we found that the inhibition of mTOR signaling pathway might cooperate in CREBZF-induced cell apoptosis in ovarian granulosa cells. mTOR is a main negative regulator of autophagy that inhibits the activation of ULK1 and ATG13 [50]. However, the activation of CREBZF caused a significant decrease of pS6K1, a marker of mTOR activation. We speculated that autophagy might participate in CREBZF-induced cell apoptosis of granulosa cells. In this study, we found that the activation of CREBZF increased the expression of LC3-II, a marker of autophagy activation. Moreover, the activation of CREBZF significantly downregulated BCL-2. It is well known that BCL-2 inhibits ATG6 (BECLIN1)-dependent autophagy. Consistent with the study that CREBZF simultaneously enhanced autophagy and apoptosis in ONS-76 cells [24], our results suggest that CREBZF-induced autophagy and apoptosis might occur in granulosa cells, with a crosstalk between these two programmed cellular death pathways.

## 4. Materials and Methods

### 4.1. Animals and Ovaries Collection

Female Kunming White mice were purchased from the laboratory animal center of the Forth Military Medicine University (Xi’an, Shanxi, China). The mice were housed separately under controlled conditions of relatively constant temperature (23 ± 2 °C) and a 12 h light–dark cycle and were provided food and water ad libitum. All animal work in this study was approved by the Experiment Center of Northwest A&F University and was in accordance with the Ethics on Animal Care guidelines for the use of animals in experimental research (Approval ID: 2016ZX08008002; date: 2 August 2016).

Mature female mice (8-weeks-old) were purchased to determine the estrous cycle by carrying out vaginal smears. Only mice that exhibited a regular 4- or 5-day estrous cycles were used for the following experiments. Immature female mice (21-day-old) were intraperitoneally injected with 5 IU of pregnant mare serum gonadotropin (PMSG, Sansheng Inc., Ningbo, China) to stimulate follicular growth for 12, 24, and 48 h, respectively. After treatment with PMSG for 48 h, the mice were intraperitoneally injected with 5 IU of human chorionic gonadotropin (hCG, Sansheng Inc., Ningbo, China) to induce ovulation and luteinization for 12, 24, and 48 h, respectively. The ovaries were excised, collected, and fixed in a 4% paraformaldehyde (PFA) solution for immunohistochemistry or stored in liquid nitrogen until use for western blot analysis.

### 4.2. Primary Mouse Ovarian Granulosa Cells Culture

Immature female mice (21-day-old) were intraperitoneally injected with 5 IU PMSG to stimulate follicular growth for 48 h. After superovulation, the ovaries were collected to culture granulosa cells in vitro. The ovaries were carefully excised, and the granulosa cells were released by puncturing the follicles with 26-gauge needles. The granulosa cells were collected via brief filtration and centrifugation, the viability of granulosa cells was determined using trypan blue dye exclusion, and the percentage of cell viability was about 85%. The cells were divided into 6-well culture plates (5 × 10^5^ per well) and cultured in Dulbecco’s modified Eagle’s medium: nutrient mixture F-12 (DMEM/F12, HyClone, Beijing, China) that was supplemented with 10% fetal bovine serum (FBS, Invitrogen, Carlsbad, CA, USA), 100 units/mL penicillin, and 100 μg/mL streptomycin solutions at 37 °C in a 5% CO_2_ atmosphere. To identify the purity of granulosa cells, we stained the adherent cells with the granulosa cell marker aromatase.

### 4.3. CREBZF Overexpression or shRNAs Lentivirus Transduction

Lentivirus vectors for CREBZF overexpression and shCREBZF were constructed by our group [27]. The recombinant lentivirus vectors were packaged and transfected into HEK 293T cells. The medium was harvested after 48 h, purified via a low-speed centrifugation, and filtered via a 0.45 µm polyvinylidene fluoride (PVDF) filter. The viral titers (TU/mL) were calculated according to the following formula: number of GFP-positive cells × multiple dilution/the amount of virus solution (mL). An appropriate number of lentiviral particles (MOI = 20) were transduced into primary granulosa cells using 8 µg/mL polybrene (Sigma Aldrich, St. Louis, MO, USA). After 12 h of incubation, the medium containing the lentivirus was removed and replaced with fresh culture medium. The cells were harvested after additional 48 h.

### 4.4. Immunohistochemistry

Immunohistochemistry was used to localize CREBZF in the ovaries. The ovaries were fixed in 4% PFA for 24 h, dehydrated through a graded ethanol series, and embedded in paraffin. Sections (5 µm thick) were mounted onto glass slides and incubated overnight at 37 °C. After hydration, the samples were placed in citrate buffer (pH = 6.0). Antigen retrieval was performed by treating the samples in a microwave oven at 92 °C for 5 min; the slides were then cooled and washed three times with PBS (pH = 7.4). The sections were pretreated with 0.3% (*v*/*v*) H_2_O_2_ in methanol to quench endogenous peroxidase activity. After washing with PBS, the sections were incubated with 10% goat serum for 30 min at 37 °C. After blocking, the sections were incubated overnight at 4 °C with an anti-CREBZF primary antibody (1:500, ab28700, Abcam, Cambridge, MA, USA). After washing followed by incubation with biotinylated anti-rabbit IgG antibodies at 37 °C for 1 h, the sections were incubated with HRP-labeled streptavidin (SA-HRP) at 37 °C for 30 min. Thereafter, positive reactions were visualized with a diaminobenzidine (DAB, Sigma-Aldrich, St. Louis, MO, USA)-peroxidase substrate and a 30 s counterstaining with hematoxylin. Finally, the sections were dehydrated and mounted. The negative control slides were incubated with pre-immune serum instead of the primary antibody. The slides were imaged using a digital microscope (BA400, Motic, Amoy, China). The intensity of immunostaining was analyzed via Image-Pro Plus Image Analysis Software (Media Cybernetics Inc., Rockville, MD, USA).

### 4.5. Immunofluorescence Staining

The granulosa cells were cultured in 24-well slide chambers for 48 h. Immunofluorescence staining of aromatase and CREBZF was performed. The granulosa cells were fixed in 4% paraformaldehyde for 10 min and permeabilized with 0.1% Triton X-100 in PBS for 10 min. After blocking with 5% BSA in PBS for 1 h at room temperature, the cells were incubated with primary antibodies to aromatase (Abcam, Cambridge, MA, USA) and CREBZF for 1 h. After washing, the cells were incubated with anti-rabbit secondary antibodies (1:500, A21206, Invitrogen) for 1 h. The nuclei were stained with 4′,6-diamidino-2-phenylindole (DAPI) for 5 min. The fluorescent signals were examined under a laser-scanning confocal microscopy (Nikon, Tokyo, Japan).

### 4.6. RNA Isolation and Quantitative Real-Time PCR Analysis

Total RNA was extracted from the frozen ovaries and granulosa cells using Trizol (TaKaRa Bio, Inc., Dalian, China) according to the manufacturer’s instructions. The cDNAs were synthesized using a PrimeScript^TM^ RT Reagent Kit (TaKaRa Bio, Inc., Dalian, China). Quantitative real-time PCR (qRT-PCR) was performed using Bio-Rad iQ5 and Bio-Rad iQ5 Optical System Software (iQ5, Bio-Rad Laboratories Inc., Hercules, CA, USA), using the SYBR Premix Ex Taq II Kit (TaKaRa Bio, Inc., Dalian, China) according to the manufacturer’s protocols. The sequences of the specific primers used are as follows: CREBZF (NM_145151.3), forward primer 5′-TAATCGGCTCAAGA AGAAGG-3′ and reverse primer 5′-CGTAGGTAGCGACTCTCC-3′; BCL-2 (NM_009741.4), forward primer 5′-CGAGAAGAAGAGGGAATCACAGG-3′ and reverse primer 5′-AATCCGTAGGAATCCCAAC C-3′; BAX (NM_007527.3), forward primer 5′-AGGATGCGTCCACCAAGAA-3′ and reverse primer 5′-CAAAGTAGAAGAGGGCAACCAC-3′; Caspase-3 (NM_001284409.1), forward primer 5′-TGACTGGAAAGCCGAAACTC-3′ and reverse primer 5′-GCAAGCCATCTCCTCATCAG-3′; and β-actin (NM_007393) forward primer 5′-GCAAGCAGGAACGATGAG-3′ and reverse primer 5′-CCATGCCAATGTTGTCTCTT-3′. These reactions were repeated thrice for each sample as technical replicates. Gene mRNA quantifications were performed using the 2^−ΔΔ*C*t^ method, and the amount of transcript in each sample was normalized using β-actin as an internal control gene to correct for differences in the cDNAs used.

### 4.7. Western Blot Analysis

Total proteins of ovaries or granulosa cells were extracted using the Total Protein Extraction Kit (Nanjing KeyGen Biotech Co., Ltd., Nanjing, China). After incubation on ice for 10 min, the proteins were precleared via centrifugation at 14,000 rpm for 15 min. Protein concentration was determined using the BCA assay (Nanjing KeyGen Biotech Co., Ltd., Nanjing, China). Equal amounts of total proteins (30 µg) were loaded into single wells and fractionated via electrophoresis in a 12% SDS-PAGE gel. The proteins were electrotransferred to PVDF membranes (Millipore, Bedford, MA, USA). After incubation in blocking solution containing 10% nonfat milk in Tris-buffered saline (TBS; pH = 7.4) and 0.5% Tween-20, the PVDF membranes were incubated overnight at 4 °C with anti-CREBZF primary antibodies and anti-β-actin mouse monoclonal antibodies (1:1000; Beijing CWBIO Co., Ltd., Beijing, China) as a loading control. The following day, the membranes were washed and incubated with biotinylated secondary antibodies (1:5000; Zhongshan Golden Bridge Bio., Nanjing, China) for 1 h at room temperature. Finally, the immunoreactive bands were visualized via ECL using the Gel Image System (Tannon Bio., Shanghai, China) and digitized with the software Quantity One (Bio-Rad Laboratories, Hercules, CA, USA). The density of the bands was quantified with the software Quantity One (Bio-Rad Laboratories, Hercules, CA, USA).

### 4.8. Apoptosis Analysis

After transduction, the apoptotic cells were quantified with an Annexin V-PE and 7-AAD apoptosis detection kit (Nanjing KeyGen Biotech Co., Ltd., Nanjing, China). The cells were washed with cold PBS, trypsinized, and harvested via centrifugation at 1000 rpm for 3 min. The cells were resuspended in 500 μL of binding buffer, followed by the addition of 5 μL of 7-AAD and 1 μL of Annexin V-PE, and incubated for 15 min. The apoptotic rate was detected by flow cytometry (EPICS Altra, Beckman Coulter Cytomics Altra) within 1 h. The experiments were independently repeated three times.

### 4.9. Statistical Analyses

The experimental results are presented as the mean ± SEM of triplicate experiments. The data were analyzed using one-way analysis of variance (ANOVA), followed by Fisher’s least significant difference test (Fisher LSD) and the Independent-Samples *T* test using the Statistical Package for the Social Sciences (SPSS) software (Version 13.0; SPSS, Inc., Chicago, IL, USA). Differences were considered significant when *p* < 0.05.

## 5. Conclusions

In summary, we detected the expression of CREBZF in the ovary and demonstrated that the main CREBZF isoform was SMILE during the estrous cycle and the treatment with PMSG and hCG in vivo. One of the main stained areas by immunohistochemistry contained a layer of ovarian granulosa cells. CREBZF induced apoptosis when overexpressed through lentivirus transduction in primary granulosa cells in vitro. As to the mechanisms, CREBZF increased BAX and cleaved Caspase-3, while it decreased BCL-2. We also found that CREBZF inhibited ERK1/2 and mTOR signaling pathways by decreasing the phosphorylation of ERK1/2 and S6K1. Taken together, this study shows that mTOR- and ERK1/2-dependent signaling pathways and caspase-dependent apoptotic pathway might cooperate in CREBZF-induced cell apoptosis in ovarian granulosa cells.

## Supplementary Materials

Supplementary materials can be found at http://www.mdpi.com/1422-0067/19/11/3517/s1. Figure S1: Localization and expression of CREBZF protein in atretic follicles of the mouse ovaries during the estrous cycle. Figure S2: Detection of pure primary granulosa cells. Figure S3: Effects of CREBZF overexpression and knockdown on the expression of apoptosis-related genes in ovarian granulosa cells.

## Abbreviations

ERK1/2	intracellular-regulated kinases 1/2
S6K1	p70 S6 kinase
CL	corpus luteum
bZIP	basic leucine zipper
CREB	cAMP response element-binding protein
ATF	activating transcription factor
HSV-1	herpes simplex virus-1
HCF-1	host cell factor 1
ER	endoplasmic reticulum
UPR	unfolded protein response
MAPK	mitogen-activated protein kinases
ATG	autophagy response genes
PMSG	pregnant mare serum gonadotropin
hCG	human chorionic gonadotropin
FBS	fetal bovine serum
PVDF	polyvinylidene fluoride
DAB	diaminobenzidine
DAPI	4′,6-diamidino-2-phenylindole
