# Supplementary material for: Activation of CREBZF Increases Cell Apoptosis in Mouse Ovarian Granulosa Cells by Regulating the ERK1/2 and mTOR Signaling Pathways
Source: Int J Mol Sci. 2018 Nov 8;19(11):3517. doi: 10.3390/ijms19113517 (PMC6274897; doi:10.3390/ijms19113517)
Supplement: Supplementary file 1 [file ijms-19-03517-s001.pdf]

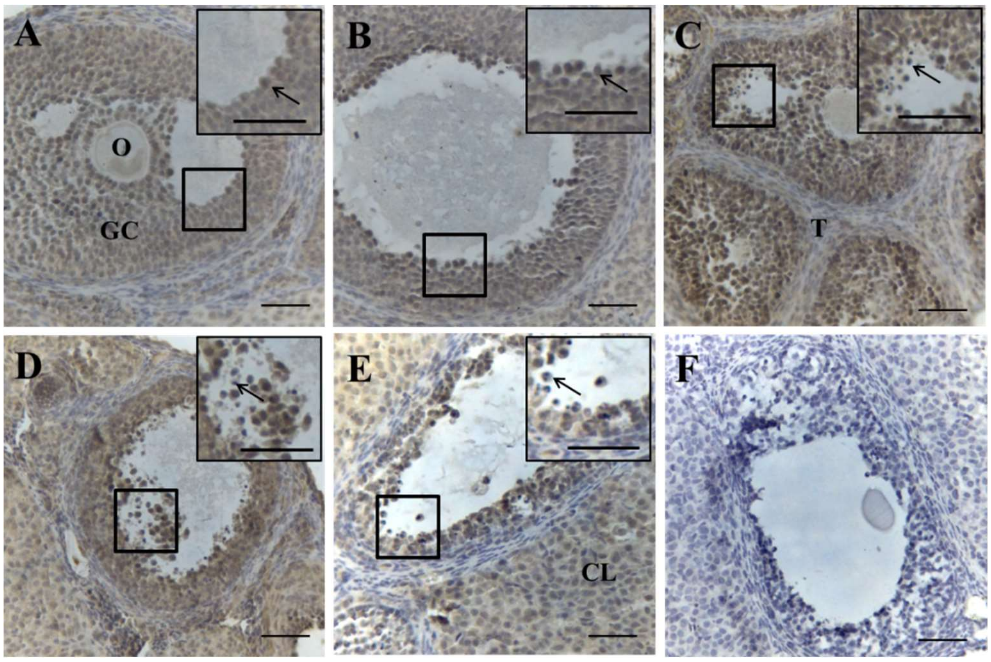

Figure S1. Localization and expression of CREBZF protein in the healthy and atretic follicles of the mouse ovaries during the estrous cycle. (**A**,**B**) Healthy antral follicles; (**C**) Early atretic follicles; (**D**) Progressed atretic follicles; (**E**) Late atretic follicles; (**F**) Negative control. The positive immunostaining for CREBZF is indicated by a brown reaction product. The nuclear of apoptotic granulosa cells was stained deep blue by hematoxylin. The upper right corner in each graph shows the enlargement of the selected area. The apoptotic granulosa cells are labeled with black arrow. GC, granulosa cells; O, oocyte; T, theca cells; CL, corpus luteum. Scale bars, 50 μm.

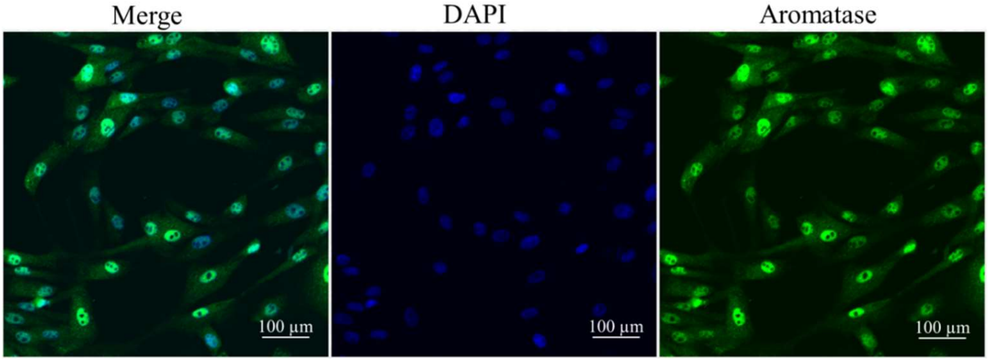

Figure S2. Detection of pure primary granulosa cells. Immunofluorescence staining analysis of primary granulosa cells was performed using antibodies against Aromatase. Scale bars, 100 μm.

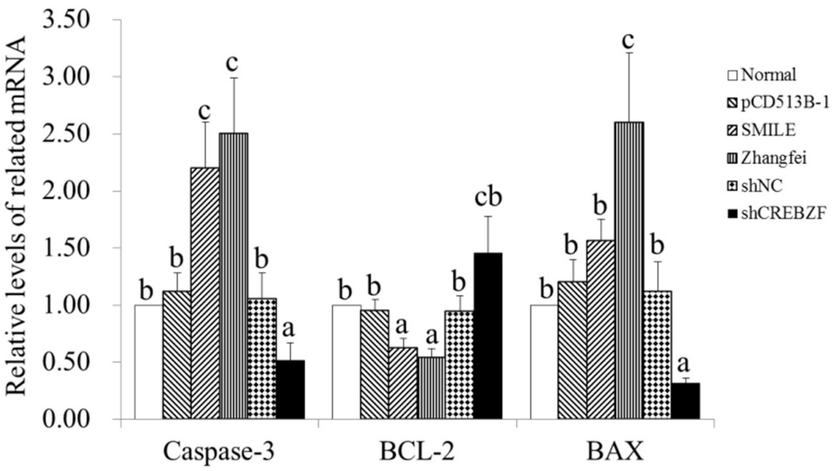

Figure S3. Effects of CREBZF overexpression and knockdown on the expression of apoptosis-related genes in the ovarian granulosa cells. The expression of cell apoptosis-related genes (Caspase-3, BCL-2 and BAX) in the ovarian granulosa cells transduced with CREBZF lentivirus for 48 h. The statistical analysis is shown in the bar graphs. Data are presented as the mean ± SEM. Bars with different letters are significantly different ($p < 0.05$).
